# Epigeneitc silencing of ribosomal RNA genes by Mybbp1a

**DOI:** 10.1186/1423-0127-19-57

**Published:** 2012-06-11

**Authors:** Bertrand Chin-Ming Tan, Chang-Ching Yang, Chia-Ling Hsieh, Yin-Hsiang Chou, Chang-Zheng Zhong, Benjamin Yat-Ming Yung, Hsuan Liu

**Affiliations:** 1 Graduate Institute of Biomedical Sciences and Department of Biomedical Sciences, College of Medicine, Chang Gung University, Kwei-San, Tao-Yuan, 333, Taiwan; 2 Department of Health Technology and Informatics, The Hong Kong Polytechnic University, Kowloon, Hong Kong; 3 Molecular Medicine Research Center, Chang Gung University, Kwei-San, Tao-Yuan, 333, Taiwan

## Abstract

**Background:**

Transcription of the ribosomal RNA gene repeats by Pol I occurs in the nucleolus and is a fundamental step in ribosome biogenesis and protein translation. Due to tight coordination between ribosome biogenesis and cell proliferation, transcription of rRNA and stable maintenance of rDNA clusters are thought to be under intricate control by intercalated mechanisms, particularly at the epigenetic level.

**Methods and Results:**

Here we identify the nucleolar protein Myb-binding protein 1a (Mybbp1a) as a novel negative regulator of rRNA expression. Suppression of rDNA transcription by Mybbp1a was linked to promoter regulation as illustrated by its binding to the chromatin around the hypermethylated, inactive rDNA gene promoters. Our data further showed that downregulation of Mybbp1a abrogated the local DNA methylation levels and histone marks associated with gene silencing, and altered the promoter occupancy of various factors such UBF and HDACs, consequently leading to elevated rRNA expression. Mechanistically, we propose that Mybbp1a maintains rDNA repeats in a silenced state while in association with the negative epigenetic modifiers HDAC1/2.

**Conclusions:**

Results from our present work reveal a previously unrecognized co-repressor role of Mybbp1a in rRNA expression. They are further consistent with the scenario that Mybbp1a is an integral constituent of the rDNA epigenetic regulation that underlies the balanced state of rDNA clusters.

## Background

Myb-binding protein 1a (Mybbp1a) was originally identified as a transcription co-repressor that could bind to the negative regulatory domain (NRD) the *c-myb* protooncogene product (c-Myb) [1,2]. Mybbp1a has the LXXLL motifs that often mediate interactions between nuclear receptors and their cofactors [3]. Mybbp1a has also been shown to interact with a number of other transcription factors, including PGC-1α, RelA/p65, Prep1, Aire, and CRY1, and exert inhibitory effect on their transactivation activity [4-9]. These findings are highly suggestive of a context-dependent co-repressor function of Mybbp1a in RNA Pol II transcription. In further support of this putative role, Mybbp1a was recently identified as a component of several co-repressor and ATP-dependent chromatin remodeling complexes, including Ret-CoR and esBAF complex [10,11], that mostly contain common constituents such as HDACs. While the roles of Mybbp1a in these repressor complexes remain unclear, it may likely serve similar epigenetic and cellular functions. Importantly, Mybbp1a is also known to preferentially interact with dimethylated histone H3K9, a marker of transcriptional repression [4]. Collectively, these observations strongly implicate Mybbp1a in the epigenetic regulation of gene expression.

Mybbp1a is located mainly within the nucleolus, and possesses in its carboxyl domain basic-amino-acid repeats that are responsible for its nuclear and nucleolar localization [12]. However, the exact role of Mybbp1a in the nucleolus is largely unknown. Its yeast homologue, Pol5p, was previously reported to be required for ribosomal DNA (rDNA) transcription [13,14]. Recently, Mybbp1a was also found to associate with nucleophosmin/B23 (NPM) [15], which is a nucleolar phosphoprotein with roles in multiple steps of ribosome biogenesis, including acting as a histone chaperone for chromatin transcription by Pol I [16,17]. Based on these attributes, the aim of this study was to characterize any functional link of Mybbp1a to ribosomal RNA (rRNA) gene expression.

The nucleolus is a nuclear subcompartment in which nascent ribosomal RNAs (rRNAs) are synthesized, processed and assembled into ribosomes. Transcription of rRNAs by Pol I is a fundamental step in ribosome biogenesis and in determining the protein synthesis capacity of the cell. Cellular control of this process is thus tightly coordinated with cellular metabolism and proliferation [18]. The rRNA genes are tandemly arrayed in hundreds of copies within nucleolar organizer regions (NORs). However, both the number and the transcriptional rate of the rRNA genes actively engaged in transcription may vary in any given cell and condition, and constitute key determinant of Pol I transcription regulation [19-21]. Efficient transcription also requires a Pol I-associated multiprotein complex that encompasses selectively factor (SL)1 and upstream binding factor (UBF) [22,23].

Chromatin context represents another significant contributory factor on the status of the rDNA clusters, which can be characterized by two different types of chromatin – an open, transcriptionally active one, and a closed one with a repressive state [24]. They are further distinguishable on the basis of distinct nucleosomal positioning, histone modifications and DNA methylation. These epigenetic characteristics are mediated and controlled by the interplay of various chromatin remodelers and modifiers [19], and, particularly for the inactive rDNA gene, by a temporal order of NoRC-mediated cofactor protein binding and enzymatic events [25,26].

Results from our present work are consistent with the scenario that Mybbp1a is an integral constituent of the rDNA epigenetic regulation. Mybbp1a acts as a suppressor of rRNA transcription by binding to the chromatin around the hypermethylated, inactive rDNA gene promoters. Our data showed that Mybbp1a was important for maintaining the local DNA methylation levels and histone marks associated with gene silencing. Lack of Mybbp1a further altered the promoter occupancy of various factors such UBF and HDACs, consequently leading to elevated rRNA expression. We propose that Mybbp1a binding, in association with HDAC1/2, maintains rDNA repeats in a silenced state and thus balances the overall status of rDNA clusters.

## Results

### Mybbp1a is a repressor of ribosomal RNA gene expression

**Figure 1:**
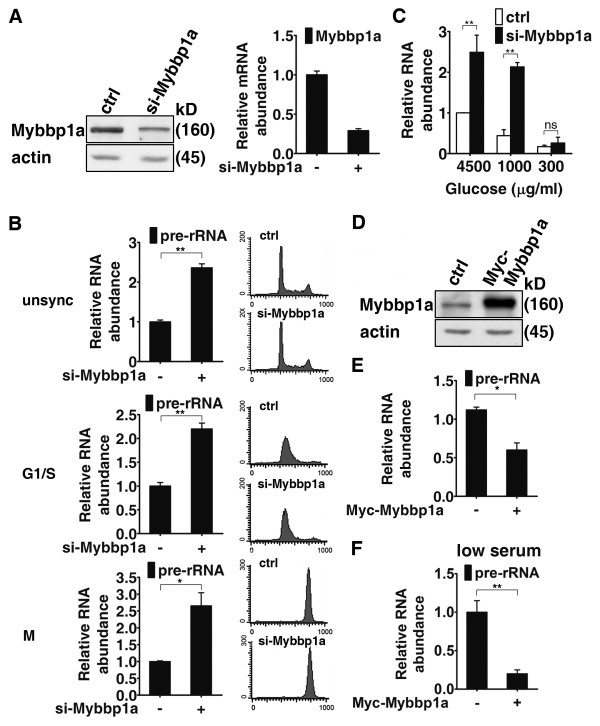
**Mybbp1a negatively regulates the expression of ribosomal RNA.** (**A**) Establishment of the control (ctrl) and Mybbp1a (si-Mybbp1a) knockdown HeLa stable lines. Expression of Mybbp1a was assessed by Western blot (left) or real-time RT-PCR (right) analysis. Actin serves as the internal control. (**B**) qRT-PCR analysis of pre-rRNA expression in control or Mybbp1a knockdown cells derived from proliferating (unsync), G1/S-arrested (G1/S), or mitotic (M) culture. (**C**) Control (ctrl) or Mybbp1a (si-Mybbp1a) knockdown cells were incubated in media containing the indicated glucose concentrations (4500, 1000, or 300 μg/ml) for 24 h. Total RNA was extracted from these cells and analyzed for pre-rRNA expression using qRT-PCR. (**D**) HeLa cells were transiently transfected with an empty vector (ctrl) or a construct encoding Myc-Mybbp1a. Expression of the ectopic Mybbp1a and actin (loading control) was examined by Western blot analysis. (**E**) & (**F**) Cells from (**D**) were cultured in either normal (**E**) or serum-free (**F**) media for 24 h, prior to qRT-PCR analysis of pre-rRNA expression. For bar graphs, data presented are normalized to GAPDH values, with the mean ± SD values from at least three experiments also shown (ns, not significant; *p < 0.05; **p < 0.01).

Given its nucleolar localization [12], association with NPM [15], and putative link to RNA Pol I in yeast [13,14], we set out to investigate the role Mybbp1a in the production of ribosomal RNA. To this end, we altered the expression of Mybbp1a by either overexpression or gene knockdown and measured the levels of pre-ribosomal RNA (pre-rRNA) by quantitative reverse transcriptase-mediated PCR. We established clones of HeLa cells stably expressing Mybbp1a-targeting shRNA (Figure 1A). Expression analysis of rRNA levels revealed significant upregulation (~2 folds) in these cells relative to the control (Figure 1B, top). Since rRNA transcription is known to be cell cycle-dependent and coupled to cell growth, we further characterized the role of Mybbp1a under different cell cycle conditions. To this end, elevation in pre-rRNA levels was similarly observed in cells at different cell cycle stages (G1/S and mitosis; Figure 1B) or under glucose/nutrient starvation (Figure 1C). Upregulation of rRNA expression in the Mybbp1a knockdown cells were similarly observed by using the Northern blot analysis (Additional file 1 Figure S1A) and nuclear run-on assay (Additional file 1 Figure S1B), further confirming the alteration in rRNA synthesis rates. Furthermore, the negative role of Mybbp1a in this functional regard was independently corroborated in a mouse myoblast cell line, C2C12 (Additional file 1 Figure S1, C & D). In contrast, transient overexpression of Mybbp1a led to a moderate but reproducible reduction in 45 S pre-rRNA levels (Figure 1, D & E, and Additional file 2 Figure S2A). Notably, a substantial decrease in nascent pre-rRNA levels was observed when Mybbp1a-expressing HeLa cells were cultured in a low serum condition (Figure 1F and Additional file 2 Figure. S2A), under which rRNA production has been shown to decrease. Together, these results imply that Mybbp1a may be a negative regulator of Pol I-mediated rRNA expression during cell growth or in the absence of mitogens.

### Mybbp1a binds to specific regions of the inactive human ribosomal DNA

**Figure 2:**
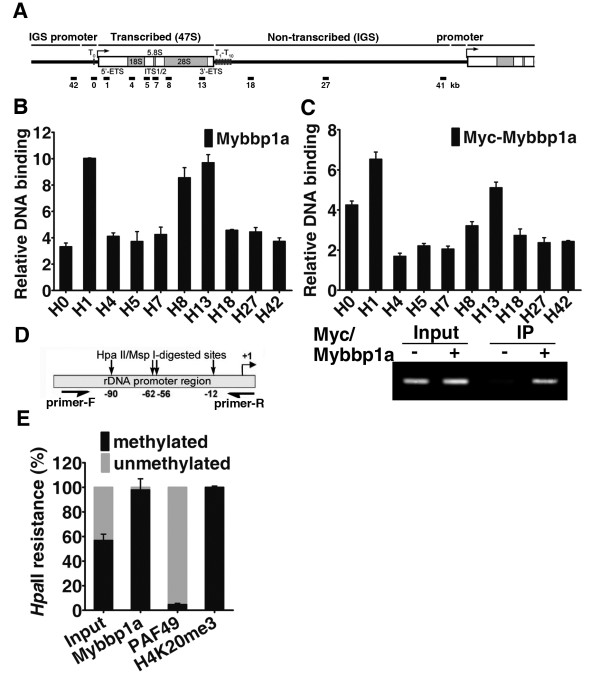
**Preferential association of Mybbp1a with chromatin regulatory region of the methylated, inactive rDNA.** (**A**) Schematic representation of a single human rDNA repeat. The gene structure and locations of the amplicons (black lines, in kb relative to the transcription start site) are shown. (**B**) & (**C**) ChIP analysis was done to profile Mybbp1a binding on the rDNA gene. Chromatin was prepared from HeLa cells (B) or HeLa cells transfected with empty vector or a Myc-tagged Mybbp1a-encoding construct (C). Enrichment of rDNA fragments precipitated with anti-Mybbp1a (B) or anti-Myc (C) antibody was quantitatively analyzed by real-time PCR using primers denoted in (A). For each amplicon, binding was normalized to the values of either IgG (B) or anti-Myc binding in the control transfection (C). ChIP, followed by end-point PCR analysis of amplicon 0 (A), was performed to confirm the binding of Mybbp1a to rDNA promoter (bottom). Representative results from one of three biological replicates are shown. (**D**) Schematic representation of the rRNA promoter region amplified in the site-specific methylation analysis and ChIP-chop assays (Primer-F and Primer-R denote respectively the forward and reverse primers) and the locations of HpaII/MspI sites (see Methods). (**E**) Quantitative analysis and graphical representation of the association of Mybbp1a, PAF49, and H4K20me3 with rDNA promoter. ChIP-chop assay was done as described in the Methods. Extent of methylation in the bound promoter rDNA is assessed by signals derived from the HpaII digestion, and is shown relative to level of unmethylated promoter.

To begin elucidating Mybbp1a function in rDNA transcription, we next explored whether the inhibitory effect of Mybbp1a occurs in the chromatin context. To do this, we performed ChIP assay to examine the association of Mybbp1a with rDNA chromatin in cells. Formaldehyde cross-linked chromatin from HeLa cells was immunoprecipitated with anti-Mybbp1a antibody. Quantitative real-time PCR using primer pair sets that span the entire human rDNA repeat was done next to obtain a high-resolution profile (~0.5-1 kb) of Mybbp1a binding throughout this locus (Figure 2A). Our results subsequently showed a distinct binding pattern, with particular enrichment at the promoter/transcription initiation site and at the end of the transcribed region (Figure 2, B). Additional ChIP experiments also pinpointed the binding of Myc-Mybbp1a to similar regions of the rDNA locus (Figure 2C). We further focused on the rDNA promoter region and confirmed binding by end-point PCR (Figure 2C, bottom).

Based on the distinct nature of rDNA repeats – the all-or-none occurrence of DNA methylation that is directly coupled to the transcriptional status [26], we next analyzed the relationship between Mybbp1a promoter occupancy and rDNA methylation. To address this issue, we performed the ChIP-chop assay that allows differentiation between the methylated and unmethylated promoter in the precipitated DNA (see Methods). In the first step, the input and precipitated DNA were digested with the isoschizomers MspI (methylation-insensitive) or HpaII (methylation-sensitive). A region of the rRNA promoter that harbors four HpaII/MspI sites was subsequently amplified by the specific primers (Figure 2D). Using quantitative real-time PCR, the HpaII-resistant PCR products generated from the input and precipitated DNA measures the level of the methylated rRNA promoter, whereas the difference between mock-digested and HpaII-digested signal reflects the level of the rRNA promoters that are unmethylated. Our results revealed that the DNA methylation levels measured at the promoter was about 60% in the HeLa cells (Figure 2E). As controls to the experiment, we also monitored methylation extent of the DNA bound by PAF49 (a subunit of RNA polymerase I) and a known repressive mark H4K20me3 (Figure 2E). As expected, PAF49 was predominantly associated with the non-methylated promoters, correlating with active transcription. Conversely, analysis of the H4K20me3 modification revealed it distribution with methylated DNA, thus correlating with inactive rRNA genes. Finally, since the rRNA promoter immunoprecipitated by anti-Mybbp1a antibody was mostly HpaII-resistant (Figure 2E), our data suggest that Mybbp1a is mainly associated with the methylated promoter. Together, these findings pinpoint the selective association of Mybbp1a with the inactive rDNA promoter in the chromatin context and correlate well with its inhibitory effect on rRNA expression.

### Mybbp1a is important for maintaining the epigenetic status of rDNA promoter

**Figure 3:**
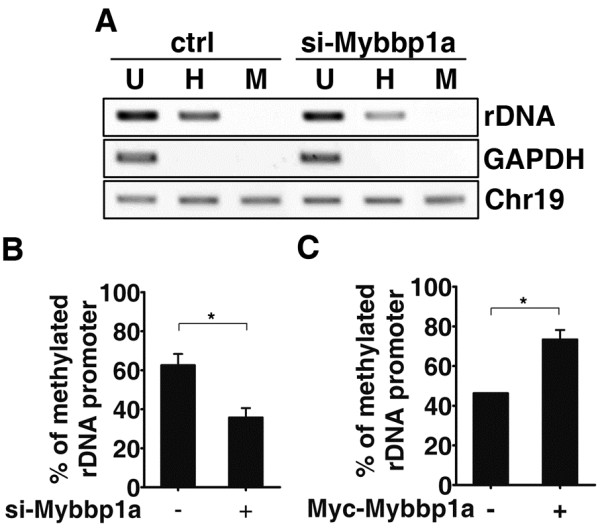
**Mybbp1a is important for maintaining rDNA promoter methylation.** (**A**) & (**B**) The rDNA promoter was amplified from undigested (U, uncut), and HpaII- (H) or MspI-digested (M) genomic DNA from control (ctrl) or Mybbp1a (si-Mybbp1a) knockdown HeLa cells, and resolved on agarose gel (A). GAPDH promoter and chromosome 19 were amplified to control for complete restriction enzyme digestion and input DNA normalization, respectively. Quantitative analysis of the results in (A) was done using real-time PCR (B). (**C**) Quantitative DNA methylation analyses were done as in (B) with genomic DNA from HeLa cells transfected with control vector (−) or Myc-Mybbp1a-encoding construct (+). (For statistical analyses shown in this figure: *p < 0.05).

Having established a potential link of Mybbp1a to the repressed rDNA genes, we next examined whether its suppression of rDNA transcription is mediated through a DNA methylation-dependent mechanism. Toward this end, we monitored the local DNA methylation status of rDNA promoter in the control vs. knockdown cells. Equivalent amounts of DNA from these cells were digested either with the HpaII or Msp1. PCR analysis, with primers that specifically amplified the region of rDNA harboring 4 HpaII/MspI sites (Figure 2D), revealed that ~60% of the rDNA promoters in the control HeLa cells were methylated (Figure 3A). However, promoter genomic DNA from Mybbp1a knockdown cells were more easily digested by HpaII, which was equivalent to a methylation rate of 36% (Figure 3A). In contrast, overexpression of Mybbp1a in HeLa cells resulted in an increase in rDNA promoter methylation as compared to the control (Figure 3B), further strengthening the notion that Mybbp1 may be required for establishing a stable DNA methylation state of the rDNA promoter region.

**Figure 4:**
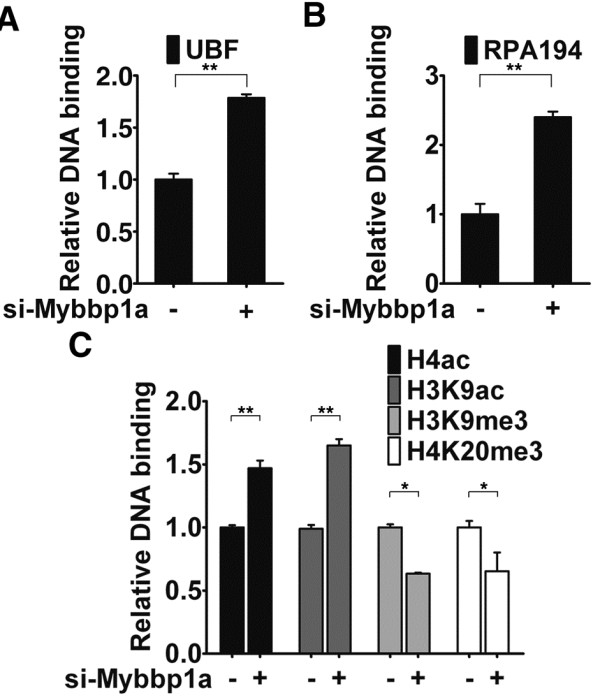
**RNAi depletion of Mybbp1a alters epigenetic status of rDNA promoters.** Mybbp1a regulates the association of RNA Pol I machinery and histone marks with rDNA promoter. Control (−) and knockdown (+) cell lines were subjected to ChIP for analyzing promoter binding of various Pol I components and epigenetic marks: UBF (**A**), RPA194 (**B**), H4ac, H3K9Ac, H3K9me3 and H4K20me3 (**C**). ChIP was carried out with control (IgG) or the specific antibodies, as denoted. Quantitative determination of the bound DNA, carried out with real-time PCR, is depicted by the bar graphs. Data presented are normalized to IgG values, with the ratio for each control group set to 1 (*p < 0.05; **p < 0.01).

Since silencing of inactive rDNA repeats is enforced by additional epigenetic repressors [27-29], we further examined Mybbp1a’s role in regulating the overall epigenetic attributes of rDNA promoter. Additional ChIP assays followed by real-time PCR were carried out on the control and Mybbp1 RNAi cells. Consistent with its function in suppressing rDNA transcription, knockdown of Mybbp1a augmented the occupancy of UBF (Figure 4A and Additional file 3 Figure S3A) and Pol I subunit RPA194 (Figure 4B and Additional file 3 Figure S3B) at the promoter and the 3’ end of the rDNA gene. Mybbp1a depletion also increased the amount of resident histone acetylation marks on H3K9 and H4, indicative of a chromatin environment accessible to transcription machinery. These observations indicate that rDNA promoter binding of RNA Pol I was promoted in the absence of Mybbp1a.

By further examining the resident histone modifications at rDNA promoter, we found that lack of Mybbp1a led to increase in the levels of H3 (H3K9) and H4 acetylation (Figure 4C). Concomitantly with the rise of activating marks, abrogation of Mybbp1a also triggered decline in the levels of rDNA promoter-associated repressive marks – H3K9me3 and H4K20me3 (Figure 4C). Collectively, these observations strongly suggest that Mybbp1a is important for the convergence of epigenetic signals at the repressed rDNA promoters and consequently the maintenance of its heterochromatic features.

### Histone deacetylase 1/2 may contribute to Mybbp1a-mediated rDNA repression

**Figure 5:**
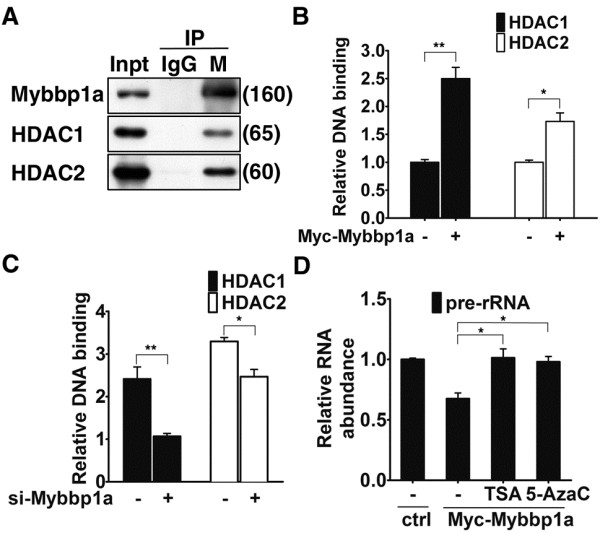
**Mybbp1a associates with and stabilizes rDNA promoter occupancy of HDAC1/2.** (**A**) Co-immunoprecipitation of HDAC1 and HDAC2 with endogenous Mybbp1a. Immunoprecipitation (IP) was done with control IgG or antibody against Mybbp1a (M). Immunoprecipiates and lysate input (Inpt, 1/40 of IP) were probed for HDAC1 and HDAC2. (**B**) & (**C**) HDAC1 and HDAC2 binding to the rRNA promoter (amplicon 0 in Figure 2A) were examined in the control (−) and Myc-Mybbp1a-overexpressing (+) HeLa cells (**B**) or the control (−) and Mybbp1a-knockdown (+) HeLa cells (**C**). Chromatin fragments were prepared from these cells and subjected to ChIP with control, HDAC1, or HDAC2 antibody. Quantitative determination of the bound DNA, carried out with real-time PCR, is shown by the bar graphs. Data presented are normalized to the IgG values, with the ratio for each control group set to 1 (*p < 0.05; **p < 0.01). (**D**) Myc-Mybbp1a-overexpressing (Myc-Mybbp1a) HeLa cells were treated with solvent (−), deacetylase inhibitor TSA, or DNMT inhibitor 5-AzaC, prior to qRT-PCR analysis of pre-rRNA expression. For bar graphs, data presented are normalized to GAPDH values, with the mean ± SD values from at least three experiments also shown (*p < 0.05).

We sought to further dissect the molecular basis of Mybbp1a’s inhibitory role in rDNA gene transcription. Given the requirement of HDACs in maintaining an inactive state of rDNA promoters [26,30], and the reported association of Mybbp1a with several HDACs-containing co-repressor complexes [10], we next aimed to assess whether HDACs contribute to Mybbp1a-mediated rDNA gene silencing. Toward this end, we first performed a co-immunoprecipitation assay and verified that there was an interaction of HDAC1 and HDAC2 with the exogeneous (Myc-Mybbp1a) or endougenous Mybbp1a (Figure 5A, and data not shown). Next, ChIP assays were used to determine whether mis-expression of Mybbp1a had an effect on HDAC occupancy of the rDNA promoter. Notably, Mybbp1a overexpression led to considerably stronger promoter binding of the HDAC enzymes (Figure 5B). Conversely, such promoter binding was reduced in the Mybbp1a knockdown cells (Figure 5C). In addition, we also demonstrated that inhibition of HDAC (by trichostatin A, or TSA) or DNMT (by 5-azacytidine, or 5-AzaC) activity reversed the negative effects of Mybbp1a overexpression on pre-rRNA expression (new Figure 5D). Collectively, these findings thus implicate Mybbp1a in facilitating or stabilizing HDACs promoter association and consequently the overall epigenetic status of the promoter chromatin.

## Discussion

In the present study we provided several lines of evidence to demonstrate a role of Mybbp1a in maintaining the silent state of the rDNA: an association with the methylated rDNA chromatin, the negative regulation of ribosomal RNA expression, and a regulatory role on the epigenetic status of the silenced rDNA promoter. Together with the previous observations that Mybbp1a is associated NPM, our data reinforced the notion that this nucleolar protein is directly involved in the production of rRNA. Our work further uncovered a novel component of the epigenetic regulatory network that underlies the balanced state of rDNA clusters.

In spite of previous reports on the potential role of Mybbp1a in gene regulation, demonstration of its functional significance in cells has been largely elusive. A recent study showed that Mybbp1a is downstream of the Aurora B kinase signaling and plays an essential role in the normal progression of mitosis [31]. Furthermore, Mybbp1a was recently found to signal a response to nucleolar stress by facilitating the p300-dependent acetylation and activation of p53 [32]. In addition, Mybbp1a has been linked, via PGC-1α and Prep1, to mitochondrial respiration and insulin-mediated glucose uptake in muscle [6,9], thus strengthening a relevant role of Mybbp1a in cellular metabolism. Collectively, these studies insinuate that Mybbp1a is an important cellular factor with pleiotropic functions. Our present study provides further support to this possibility by establishing a previously unrecognized link of the Mybbp1a protein to ribosomal RNA expression regulation. Its nucleolar function may thus underpin proper protein production and cell proliferation.

Due to tight coordination between ribosome biogenesis and cell proliferation and metabolism, transcription of rRNA and stable maintenance of rDNA clusters are thought to be under intricate control by intercalated mechanisms, particularly at the epigenetic level. Various chromatin remodeling and modifying activities with a role in rDNA transcription have been identified [19,24]. To such regulatory networks, we have added a new important one. The observed functional attributes of Mybbp1a are in accordance with those previously reported for other rDNA-associated epigenetic repressors, such as JHDM1B [33] and energy-dependent nucleolar silencing complex (eNoSC) [34]. Interestingly, an energy-sensing nucleolar pathway involving eNoSC and Mybbp1a was recently implicated in the nucleolar stress-associated regulation of p53 [35]. Possible functional and physical interaction between Mybbp1a and these factors may thus contribute to a robust and dynamic expression of the ribosomal RNA. However, while an upregulation of rRNA may be accompanied by greater extent of cellular proliferation and sometimes an increase in cell size, as shown in the case of JHDM1B, knockdown of Mybbp1a led to instead a slower cell growth and slighted delayed G1-S phase progression (data not shown). This discrepancy may be explained by the potentially multifunctional nature of Mybbp1a, abrogation of which may elicit compensatory changes that culminate in cell growth arrest and/or defect. In addition, disturbance of nucleolar content as a consequence of rRNA accumulation may also activate stress signal cascades that ultimately restrain cell growth.

While a repressor function on rDNA genes has been ascribed to Mybbp1a by our results, how such role is manifested remains an unanswered question. Mybbp1a is known to associate with diverse transcriptional regulators, acting largely as a repressor for Pol II transcription. In this capacity, Mybbp1a was recently identified as a component of several HDAC-containing co-repressor and ATP-dependent chromatin remodeling complexes, including Ret-CoR and esBAF complex [10,11]. These enzymatic activities are intimately associated with the processes of differentiation and stem cell physiology, and mostly contain common constituents such as HDACs. Unlike other chromatin remodeling factors, no enzymatic activity has been described for Mybbp1a, despite the presence of several domains that are characteristic of transcriptional regulators, such as leucine zipper-like, basic, and acidic motifs. Intriguingly, the binding of Mybbp1a with histone H3 amino terminus that is dimethylated on the Lys9 residue was demonstrated previously [4]. These findings thus imply that Mybbp1a may be capable of recognizing distinct chromatin domains, particularly the silenced regions, and consequently exerting its regulatory functions. This mode of action may resemble that recently documented for the component of eNoRC, nucleomethylin [34]. Together with the observations that abrogation of Mybbp1a impaired the rDNA promoter association of HDACs and DNA methylation, these attributes may pinpoint the repressor function of Mybbp1a temporally at a step subsequent to eNoRC but preceding or concomitant with the recruitment of HDACs and DNMT to the inactivated promoter. Furthermore, it is a formal possibility that Mybbp1a may serve as a structural adaptor for the co-repressor complexes, or an enhancer for the rDNA binding and activity of the various components.

## Methods

### Cell culture and synchronization

HeLa cells were cultured in Dulbecco’s modified Eagle’s medium (DMEM, Invitrogen) supplemented with 10% heat-inactivated fetal bovine serum (FBS, Invitrogen) and 100 U/ml penicillin and streptomycin solution (Invitrogen). Mouse C2C12 myoblast cells were cultured similarly, except with supplementation of 20% heat-inactivated FBS. Cells were maintained in a 5% CO_2_ humidified incubator at 37 °C. Cell cycle synchronization was done based on a previous report [36]. For M phase synchronization, subconfluent cells were treated with 200 ng/ml nocodazole for 10 h. Mitotic cells were harvested through detachment by mitotic shake-off. Cells were synchronized at the G1/S boundary by double thymidine block. Cell cycle profiles were monitored by FACS. For inhibition of HDAC and DNMT activity, cells were treated respectively with 200 nm of TSA and 5 μM of 5-AzaC for 24 hrs before gene expression analysis.

### RNAi and establishment of stable cell lines expressing the Mybbp1a-targeting shRNA constructs

Plasmid-based shRNA-expressing constructs (pSuper) targeting Mybbp1a (5’-GAGACCAAGAAGCGAAAGA-3’ & 5’-CTGAGTGGGAGCAGCTGAT-3’) and luciferase (control) were transfected into HeLa cells using Lipofectamine 2000 reagent (Invitrogen) to obtain stable lines. Stable clones overexpressing Mybbp1a were established using pcDNA3.1 vector encoding Myc-Mybbp1a [6]. These cells were plated at low density, subjected to drug selection with 850 μg/ml G418 (Invitrogen), and clonal colonies were isolated. Several drug-resistant C2C12 cell lines were established for each construct and pooled for further characterization. Their levels of Mybbp1a protein were determined by immunoblotting. For transient knockdown of Mybbp1a in HeLa, cells were transfected with control or a pool of two Mybbp1a-specific siRNAs with Lipofectamine RNAiMAX (Invitrogen). Twenty-five-nucleotide siRNA duplexes (Stealth, Invitrogen) were designed targeting different regions of the mRNA: +1718 to +1743 (5’-AGATGATGAGTACTCTGAAGGAATT-3’); +3135 to +3160 (5’-CCTGA TGCTCCAGAAGACTCTGTCT-3’).

### Cell lysate preparation

Cells were harvested and washed twice in PBS. Whole cell extracts were prepared using WCE buffer [20 mM HEPES, pH 7.4, 0.2 M NaCl, 0.5% Triton X-100, 10% glycerol, 1 mM EDTA, 1 mM EGTA, 10 mM β-glycerophosphate, 2 mM Na3VO4, 1 mM NaF, 1 mM DTT, cocktail protease inhibitor (Roche)]. For preparation of nuclear extracts, cell pellets were resuspended in 1 ml of lysis buffer (10 mM Tris–HCl pH 7.4, 10 mM NaCl, 3 mM MgCl_2_, 0.5% NP-40 and cocktail protease inhibitor) per 10^7^ cells. After 10-min incubation on ice, nuclei were collected by centrifugation (500 g, 5 min) and washed with lysis buffer devoid of NP-40. After centrifugation, the pellet was resuspended in 100 μl nuclei lysis buffer (10 mM Tris–HCl pH 7.4, 400 mM NaCl, 1 mM EDTA, 1 mM DTT and cocktail protease inhibitor), mixed thoroughly for 30 min at 4 °C. The nuclei lysates were diluted 10-fold in WCE buffer and centrifuged (16000 g, 20-min, 4 °C) to obtain the nuclear fraction. Lysates were boiled in 2X urea sample buffer dye (100 mM Tris–HCl, pH 6.8, 4% SDS, 0.2% bromophenol blue, 20% glycerol, 200 mM β-mercaptoethanol, 8 M urea), and fractionated by SDS-PAGE.

### Reagents and antibodies

All chemicals were purchased from sigma (St. Louis, MO), except where otherwise indicated. Mybbp1a specific antibody (Cell Signaling Technology; Danvers, MA. USA) was raised in rabbit using a recombinant protein corresponding to amino acids 1092–1214 of mouse Mybbp1a, followed by antigen-specific purification. Anti-Myc-tag monoclonal antibody was from Cell Signaling Technology (Danvers, MA. USA). β-actin specific monoclonal antibody and polyclonal antibodies against HDAC1, HDAC2, H3K9me3, H4ac were from Millipore (Temecula, CA, USA). αH3K9Ac, αH3K9Me2, αSuv39h1, αSWI/SNF, and αPCAF rabbit polyclonal antibodies were purchased from Abcam (Cambridge, MA, USA). Anti-PAF49/CAST antibody was obtained from Bethyl Laboratories (Montgomery, TX, USA). Secondary antibodies used in the Western blot assays were from Vector Laboratories (Burlingame, CA, USA), whereas those used in immunofluorescence analysis were obtained from Invitrogen.

### Western blot analysis and immunoprecipitation

Western blot analysis was performed after electrophoretic separation of polypeptides by 7.5 or 12.5% SDS-PAGE and transfer to Immobilon-P/PVDF membranes (Millipore). Blots were probed with the indicated primary and appropriate secondary antibodies. Immuno-bands were subsequently detected by the enhanced chemi-luminescence reaction (ECL) (PerkinElmer; Waltham, MA, USA). All immunoprecipitations were performed with equal amounts of cell extract protein (1 mg) incubated with the indicated antibodies (2.5 μg) at 4 °C for overnight. The immunocomplexes were captured with protein G-sepharose (Millipore) for 1 hr at 4 °C with rotation. The protein G-antigen-antibody complexes were washed six times with the WCE buffer, and boiled in 2X sample buffer dye for subsequent PAGE and immunoblotting analysis as described above.

### RNA isolation and reverse transcription (RT)-PCR

Total RNA from cells was isolated using the TRIzol reagent (Invitrogen) according to the manufacturer’s instructions. Genomic DNA was removed by digestion with 2U of DNase I (Ambion; Foster City, CA, USA). cDNA was synthesized by MMLV reverse transcriptase (Invitrogen) using random hexamers. Quantitative determination of the cDNA levels was done by real-time PCR using the Bio-Rad iQ5 Gradient Real Time SYBR-Green PCR system. Levels of cDNA were normalized to the GAPDH values of the respective samples. All results represent the mean ± SD of at least three independent experiments. Sequences of the primers are listed in Additional file 4 Table S1.

### Northern blot analysis and nuclear run-on assay

Additional methods for measuring the rRNA transcription rate – Northern blot analysis and nuclear run-on assay – were performed as essentially described elsewhere [37,38], with some modifications. For the nuclear run-on assay, we used Digitonin (40 mg/ml; Sigma) in place of NP-40 to permeabilize cells. Cells were washed twice with ice-cold 1X PBS and removed from the culture plate using a cell scraper in 1 ml of 1X PBS per 10-cm dish and collected by centrifugation (500 g, 5 min). Cell pellets were resuspended in 1 ml of lysis buffer (10 mM Tris–HCl, pH 7.4, 10 mM NaCl, 3 mM MgCl_2_, 40 mg/ml Digitonin) per 10^7^ cells. After 10-min incubation on ice, nuclei were then collected by centrifugation (500 g, 5 min) and washed with lysis buffer devoid of Digitonin. To perform run-on reactions, aliquots of nuclei were mixed with 100 ml of 2X reaction buffer (20 mM Tris–HCl pH 8.0, 5 mM MgCl_2_, 200 mM KCl, 4 mM dithiothreitol, 1 mM each of ATP, CTP and GTP, 200 mM sucrose and 20% glycerol) and biotin-16-UTP (Epicentre) in a final volume of 200 ml at 29 °C for 30 min. A total of 60U of RNase-free DNaseI (Fermentas; Burlington, Ontario, Canada) and 6 ml of 250 mM CaCl_2_ were added, and the reaction mixture was incubated for an additional 10 min at 37 °C. Biotinylated RNA was purified by Dynabeads M-280 (Invitrogen), a magnetic bead covalently linked to streptavidin. Dynabeads resuspended in binding buffer (10 mM Tris–HCl, pH 7.5, 1 mM EDTA and 2 M NaCl) were mixed to an equal volume of run-on RNA and subjected a 2-hr incubation at room temperature. Beads were separated by the magnetic apparatus and washed once with 500 ml 2X SSC-15% formamide for 10 min and twice with 1 ml 2X SSC for 5 min each. Random hexamer-primed cDNA was synthesized using 10 ml biotinylated RNA, and subsequently subjected to semi-quantitative PCR to assay for 45 S pre-rRNA transcription rate. To ensure the efficiency of the reverse transcription, the intensities of PCR products were normalized to those of GAPDH. Generation of the probe for the Northern blot analysis was based on the previous report [38].

### Chromatin immunoprecipitation (ChIP) and real-time PCR analysis

ChIP assay was performed essentially as described previously [39]. Crosslinked, sonicated chromatin was precleared before being incubated with 2.5 μg of the indicated antibodies and rotated at 4 °C overnight. Normal mouse or rabbit IgG (Millipore) was used for the mock immunoprecipitation. After extensive washes, immunocomplexes were treated with Proteinase K and decrosslinked. Bound DNA in the precipitates, as well as input DNA (1/10 fragmented chromatin), was extracted, purified, and subjected to real-time PCR analysis using primers corresponding to different regions of the rDNA repeat unit. Real-time PCR reactions were conducted on the Bio-Rad iQ5 Gradient Real Time PCR system, using the 2X SYBR Green Master mix (Bio-Rad, USA). Results were corrected for nonspecific binding to IgG and presented as percentage of input DNA. Triplicate PCRs for each sample were carried out. The ChIP primer sequences are listed in Additional file 4 Table S2.

### rDNA promoter site-specific methylation analysis

Genomic DNA was isolated from the HeLa cells by using QIAamp DNA Mini kit (QIAGEN). 5 μg of DNA were digested overnight with methylation-sensitive (HpaII) or methylation-insensitive (MspI) restriction enzymes. Subsequently, rDNA promoter (HrDNA 42.9 kb) was amplified from undigested and HpaII/MspI-digested genomic DNA. GAPDH promoter and a chromosome 19 fragment were also amplified as controls respectively for complete enzyme digestion and equal DNA input. The primer sequences for GAPDH promoter are as described elsewhere [40], and primer sequences for the human chromosome 19 region are as follows: Forward, 5’-CTATGCCAAGCCCATTCTAGGTCCT-3’; Reverse, 5’-GCAGGGAAACTGTGCACAGCAAGAG-3’. For quantitative analysis, real-time PCR was also performed.

### Methylation-sensitive ChIP-chop assay

The ChIP-chop experiment was done based on previous reports [40,41]. To distinguish between the methylated and unmethylated promoter, the input and precipitated DNA of the ChIP samples were digested with the isoschizomers MspI or HpaII prior to quantitative real-time PCR analysis. The digests along with an equal amount of the undigested immunoprecipitated DNA were amplified as described above. Oligonucleotide sequences and quantitative PCR assay characteristics are shown in Additional file 4 Table S2. The HpaII-resistant PCR product generated from the input DNA measures the level of methylated rRNA promoter, whereas the difference between mock-digested and HpaII-digested signal reflects the level of the unmethylated rRNA promoters. The results were depicted as the ratio of methylated to unmethylated DNA precipitated with the antibodies in the ChIP.

## Competing interests

The authors declare that they have no competing interests.

## Authors' contributions

BCMT and CCY, CLH and YHC performed the gene expression, DNA methylation, and ChIP experiments. CCY and CZZ carried out cell culture and manipulation. BCMT, BYMY, and HL conceived of the study, and participated in its design and coordination and helped to draft the manuscript. All authors read and approved the final manuscript.

## Supplementary Material

Additional file 1 **Figure S1.**Independent confirmation of the negative role of Mybbp1a in rRNA expression (related to Figure 1, B to F). (A) Total RNA was extracted from control and Mybbp1a knockdown (si-Mybbp1a) cells. The levels of 47 S pre-rRNA as well GAPDH (as a control) were analyzed in a northern blot probed with a dig-labeled DNA probe. (B) Nuclear run-on assay was performed as described in the Methods, on the control (−) and Mybbp1a-knockdown (+) HeLa cells. (C) & (D) Mouse C2C12 myoblast cells were transfected with control (−) or Mybbp1a-targeting (+) siRNA for 48 hrs. Total RNA was then prepared for expression analysis. Extent of Mybbp1a downregulation was assessed by real-time RT-PCR analysis (C). Expression of pre-rRNA was analyzed also by quantitative RT-PCR (D). For bar graphs, data presented are normalized to GAPDH values, with the mean ± SD values from at least three experiments also shown (**p < 0.01; ***p < 0.001). Click here for file

Additional file 2 **Figure S2.**Cell cycle profiles of the cells in Figure 1, E & F. Cells transiently harboring control (ctrl) or Myc-Mybbp1a-expression plasmid were subjected to flow cytometry analysis for measurement of DNA content. Cells in the G1, S, and G2/M phases were defined by gating. Percentages of gated events are summarized on the right. Click here for file

Additional file 3 **Figure S3.**Mybbp1a regulates the association of RNA Pol I machinery with rDNA gene (related to Figure 4, A & B). Control (ctrl) and knockdown (si-Mybbp1a) cell lines were subjected to ChIP for analyzing promoter binding of UBF (A) and RPA194 (B). ChIP was carried out with control (IgG) or the specific antibodies, as denoted. Quantitative determination of the bound DNA, carried out with real-time PCR, is depicted by the bar graphs. Primers corresponding to various regions of the rDNA gene, as denoted in Figure 2A, were used. Data presented are normalized to IgG values, with the ratio for each control group set to 1 (ns, not significant; *p < 0.05; **p < 0.01; ***p < 0.001). Click here for file

Additional file 4 **Table S1.** Primers for quantitative RT-PCR. Table S2. Primers for ChIP assay. Click here for file
