# Supplementary figures and images for: Epigeneitc silencing of ribosomal RNA genes by Mybbp1a
Source: J Biomed Sci. 2012 Jun 11;19(1):57. doi: 10.1186/1423-0127-19-57 (PMC3407492; doi:10.1186/1423-0127-19-57)

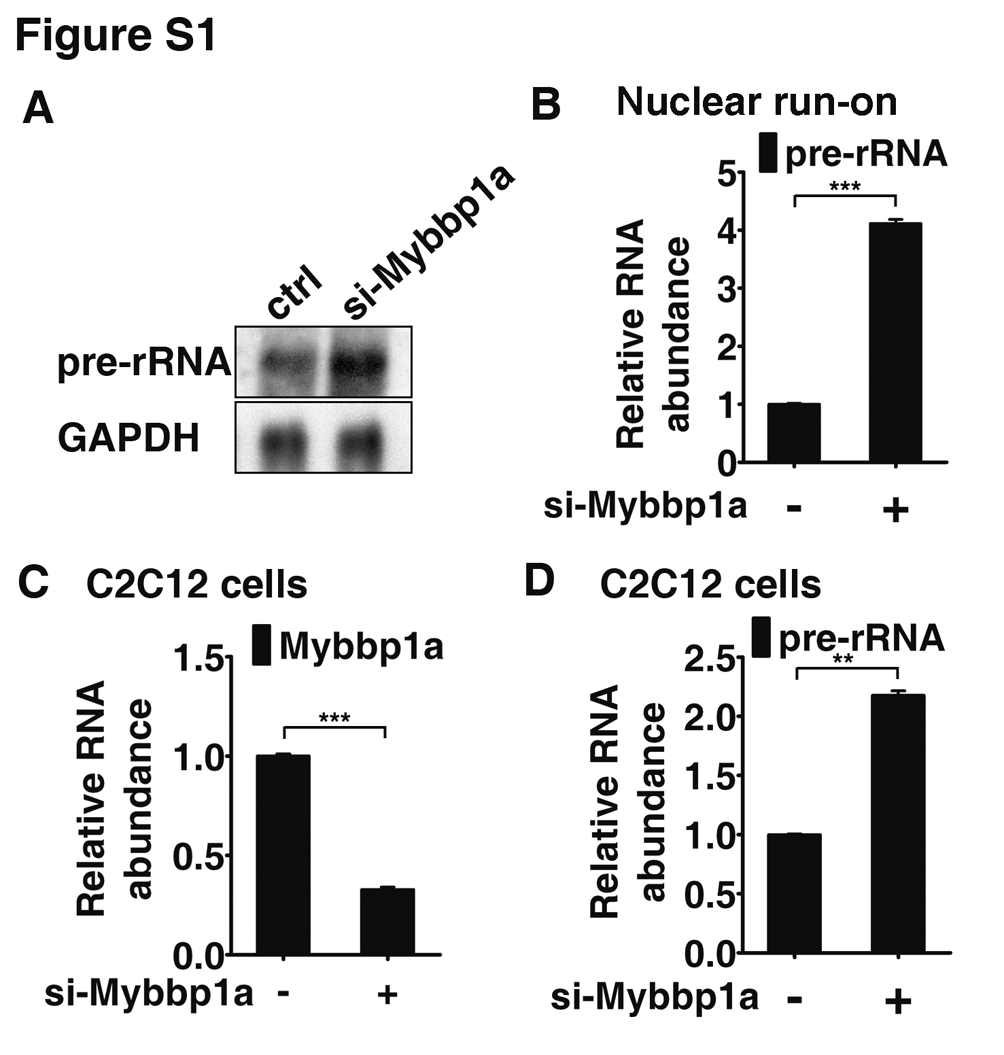

Supplement: Additional file 1 — Figure S1.Independent confirmation of the negative role of Mybbp1a in rRNA expression (related to Figure 1, B to F). (A) Total RNA was extracted from control and Mybbp1a knockdown (si-Mybbp1a) cells. The levels of 47 S pre-rRNA as well GAPDH (as a control) were analyzed in a northern blot probed with a dig-labeled DNA probe. (B) Nuclear run-on assay was performed as described in the Methods, on the control (−) and Mybbp1a-knockdown (+) HeLa cells. (C) & (D) Mouse C2C12 myoblast cells were transfected with control (−) or Mybbp1a-targeting (+) siRNA for 48 hrs. Total RNA was then prepared for expression analysis. Extent of Mybbp1a downregulation was assessed by real-time RT-PCR analysis (C). Expression of pre-rRNA was analyzed also by quantitative RT-PCR (D). For bar graphs, data presented are normalized to GAPDH values, with the mean ± SD values from at least three experiments also shown (**p < 0.01; ***p < 0.001). [file 1423-0127-19-57-S1.tiff]

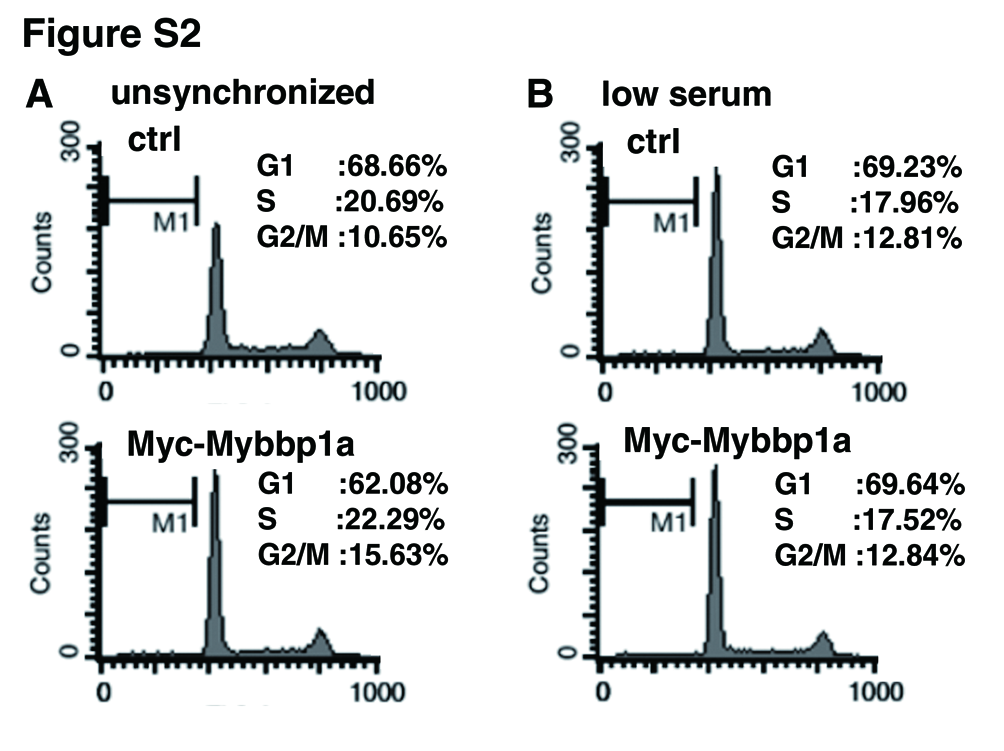

Supplement: Additional file 2 — Figure S2.Cell cycle profiles of the cells in Figure 1, E & F. Cells transiently harboring control (ctrl) or Myc-Mybbp1a-expression plasmid were subjected to flow cytometry analysis for measurement of DNA content. Cells in the G1, S, and G2/M phases were defined by gating. Percentages of gated events are summarized on the right. [file 1423-0127-19-57-S2.tiff]

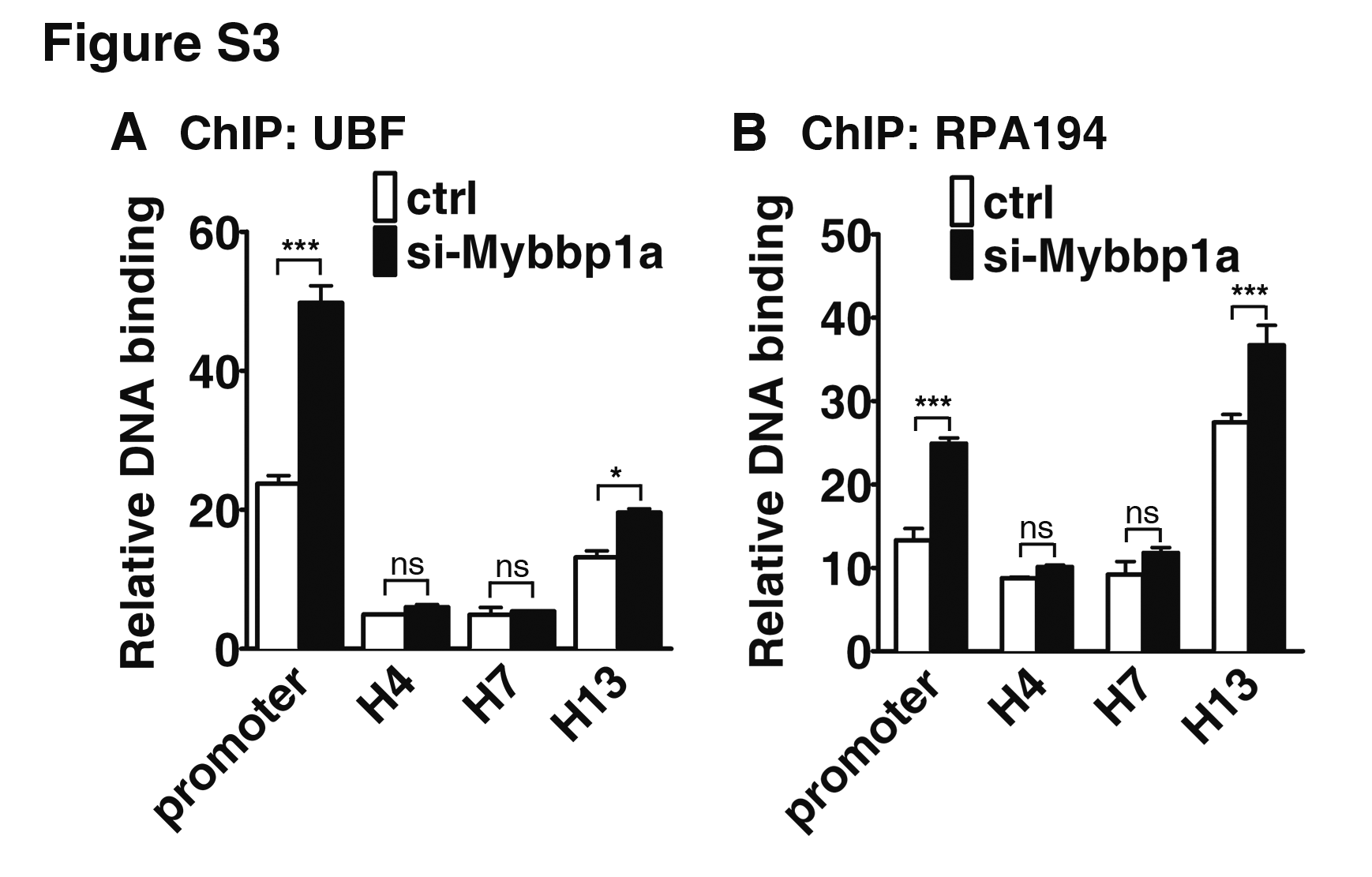

Supplement: Additional file 3 — Figure S3.Mybbp1a regulates the association of RNA Pol I machinery with rDNA gene (related to Figure 4, A & B). Control (ctrl) and knockdown (si-Mybbp1a) cell lines were subjected to ChIP for analyzing promoter binding of UBF (A) and RPA194 (B). ChIP was carried out with control (IgG) or the specific antibodies, as denoted. Quantitative determination of the bound DNA, carried out with real-time PCR, is depicted by the bar graphs. Primers corresponding to various regions of the rDNA gene, as denoted in Figure 2A, were used. Data presented are normalized to IgG values, with the ratio for each control group set to 1 (ns, not significant; *p < 0.05; **p < 0.01; ***p < 0.001). [file 1423-0127-19-57-S3.tiff]
