# Supplementary material for: Epigeneitc silencing of ribosomal RNA genes by Mybbp1a
Source: J Biomed Sci. 2012 Jun 11;19(1):57. doi: 10.1186/1423-0127-19-57 (PMC3407492; doi:10.1186/1423-0127-19-57)
Supplement: Additional file 4 — Table S1. Primers for quantitative RT-PCR. Table S2. Primers for ChIP assay. [file 1423-0127-19-57-S4.docx]

**Supplementary Information (Tan et al.)**

**Table S1. Primers for quantitative RT-PCR**

| **Name** | **Orientation** | **Sequence (5’ 🡪 3’)** |
| --- | --- | --- |
| 47S pre-rRNA | Forward | GCT GAC ACG CTG TCC TCT GG |
|  | Reverse | GAG AAC GCC TGA CAC GCA CG |
| 45S pre-rRNA | Forward | GCC TTC TCT AGC GAT CTG AGA G |
|  | Reverse | CCA TAA CGG AGG CAG AGA CA |
| NPM | Forward | AAC TTG CTG CTG ATG AAG AT |
|  | Reverse | TTG ACT TTT GTG CAT TTT TG |
| Mybbp1a | Forward | GAC TTC TTC TGG GAC ATT GC |
|  | Reverse | CGC TTC AGG GCA TAT TTC AT |
| GAPDH | Forward | GGT ATC GTG GAA GGA CTC ATG AC |
|  | Reverse | GCT GAA CGG GAA GCT CAC TGG CAT |

**Table S2. Primers for ChIP assay**

| **HrDNA (in kb)** | **Orientation** | **Sequence (5’ 🡪 3’)** |
| --- | --- | --- |
| 42.9 (promoter) | Forward | CTG CGA TGG TGG CGT TTT TG |
|  | Reverse | ACA GCG TGT CAG CAT ATA ACC |
| 1.4 (5’ETS) | Forward | GCC TTC TCT AGC GAT CTG AGA G |
|  | Reverse | CCA TAA CGG AGG CAG AGA CA |
| 4.1 (18S) | Forward | AAC GGC TAC CAC ATC CAA GG |
|  | Reverse | GGG AGT GGG TAA TTT GCG C |
| 5.3 (ITS1) | Forward | AGT GCG GGT CAT AAG CTT GC |
|  | Reverse | GGT GTG TAC AAA GGG CAG GG |
| 6.6 (5.8S) | Forward | CTC TTA GCG GTG GAT CAC TCG |
|  | Reverse | GCT AGC TGC GTT CTT CAT CGA |
| 8 (28S) | Forward | AGT CGG GTT GCT TGG GAA TGC |
|  | Reverse | CCC TTA CGG TAC TTG TTG ACT |
| 13 (28S) | Forward | ACC TGG CGC TAA ACC ATT CGT |
|  | Reverse | GGA CAA ACC CTT GTG TCG AGG |
| 18 (IGS) | Forward | GTT GAC GTA CAG GGT GGA CTG |
|  | Reverse | GGA AGT TGT CTT CAC GCC TGA |
| 27 (IGS) | Forward | CCT TCC ACG AGA GTG AGA AGC G |
|  | Reverse | CTC GAC CTC CCG AAA TCG TAC A |
| 41 (IGS) | Forward | ACG TTT CTG TAC GCT TAT ATG CAA A |
|  | Reverse | AAT GCA GAG ATA CAC GTT GTC G |

**Supplementary Figure Legends**

**Figure S1. Independent confirmation of the negative role of Mybbp1a in rRNA expression (related to Figure 1, B to F).**

(A) Total RNA was extracted from control and Mybbp1a knockdown (si-Mybbp1a) cells. The levels of 47S pre-rRNA as well GAPDH (as a control) were analyzed in a northern blot probed with a dig-labeled DNA probe. (B) Nuclear run-on assay was performed as described in the Supplementary Methods, on the control (−) and Mybbp1a-knockdown (+) HeLa cells. (C) & (D) Mouse C2C12 myoblast cells were transfected with control (-) or Mybbp1a-targeting (+) siRNA for 48 hrs. Total RNA was then prepared for expression analysis. Extent of Mybbp1a downregulation was assessed by real-time RT-PCR analysis (C). Expression of pre-rRNA was analyzed also by quantitative RT-PCR (D). For bar graphs, data presented are normalized to GAPDH values, with the mean ± SD values from at least three experiments also shown (***p* < 0.01; ****p* < 0.001).

**Figure S2. Cell cycle profiles of the cells in Figure 1, E & F.**

Cells transiently harboring control (ctrl) or Myc-Mybbp1a-expression plasmid were subjected to flow cytometry analysis for measurement of DNA content. Cells in the G1, S, and G2/M phases were defined by gating. Percentages of gated events are summarized on the right.

**Figure S3. Mybbp1a regulates the association of RNA Pol I machinery with rDNA gene (related to Figure 4, A & B).**

Control (ctrl) and knockdown (si-Mybbp1a) cell lines were subjected to ChIP for analyzing promoter binding of UBF (A) and RPA194 (B). ChIP was carried out with control (IgG) or the specific antibodies, as denoted. Quantitative determination of the bound DNA, carried out with real-time PCR, is depicted by the bar graphs. Primers corresponding to various regions of the rDNA gene, as denoted in Figure 2A, were used. Data presented are normalized to IgG values, with the ratio for each control group set to 1 (ns, not significant; **p* < 0.05; ***p* < 0.01; ****p* < 0.001).
